# Calpain-10 Expression Is Elevated in Pancreatic Islets from Patients with Type 2 Diabetes

**DOI:** 10.1371/journal.pone.0006558

**Published:** 2009-08-18

**Authors:** Charlotte Ling, Leif Groop, Silvia Del Guerra, Roberto Lupi

**Affiliations:** 1 Department of Clinical Sciences, Diabetes and Endocrinology, Lund University, Clinical Research Centre (CRC), Malmö, Sweden; 2 Department of Endocrinology and Metabolism, Metabolic Unit, University of Pisa, Pisa, Italy; University of Bremen, Germany

## Abstract

**Background:**

Calpain-10 was the first gene to be identified influencing the risk of type 2 diabetes (T2D) by positioning cloning. Studies in β-cell lines and rodent islets suggest that calpain-10 may act as a regulator of insulin secretion. However, its role in human pancreatic islets remains unclear. The aim of this study was to examine if calpain-10 expression is altered in islets from patients with T2D and if the transcript level correlates with insulin release. We also tested if polymorphisms in the *CAPN10* gene are associated with gene expression and insulin secretion in vitro.

**Methodology/Principal Findings:**

Calpain-10 mRNA expression was analysed in human pancreatic islets from 34 non-diabetic and 10 T2D multi-organ donors. *CAPN10* SNP-43 and SNP-44 were genotyped and related to gene expression and insulin release in response to glucose, arginine and glibenclamide. The mRNA level of calpain-10 was elevated by 64% in pancreatic islets from patients with T2D compared with non-diabetic donors (*P* = 0.01). Moreover, the calpain-10 expression correlated positively with arginine-stimulated insulin release in islets from non-diabetic donors (*r* = 0.45, *P* = 0.015). However, this correlation was lost in islets from patients with T2D (*r* = 0.09; *P* = 0.8). The G/G variant of SNP-43 was associated with reduced insulin release in response to glucose (*P*≤0.04) in non-diabetic donors.

**Conclusions:**

While calpain-10 expression correlates with insulin release in non-diabetic human islets, this correlation is lost in T2D suggesting that a stimulatory effect of calpain-10 could be lost in patients with T2D.

## Introduction

Impaired insulin secretion from pancreatic β-cells together with insulin resistance in peripheral tissues represent hallmarks of type 2 diabetes (T2D). The disease develops as a conspiracy between the environment and the genetic background. Indeed, common variants in a number of genes have recently been associated with T2D [1]. However, the first gene to be linked to T2D by positional cloning was *CAPN10* encoding for a cystein protease, calpain-10 [2] and polymorphisms in this gene have been associated with T2D in some but not all populations [3], [4], [5], [6]. Calpains are calcium-dependent intracellular nonlysosomal proteases that can hydrolyze substrates important in calcium-regulated signaling pathways [7]. Calpain-10 has been suggested to influence both insulin secretion and resistance [8], [9], [10], [11], [12], [13], [14], [15], [16]. Furthermore, common variants in the *CAPN10* gene have been associated with transcript levels in skeletal muscle and adipose tissue [9], [11], [17]. Although great efforts have been made to understand the role of calpain-10 in T2D its function and regulation in human pancreatic islets remain to be determined. The aims of the present study were therefore to 1) analyze calpain-10 expression in pancreatic islets from multi-organ donors with and without T2D, 2) examine if two polymorphisms (SNP-43 and SNP-44) in the *CAPN10* gene are associated with gene expression and insulin secretion in human islets and 3) test if calpain-10 expression is associated with insulin secretion in human islets.

## Methods

### Subjects

The characteristics of the non-diabetic and T2D multi-organ donors, whose pancreases have been processed for islet preparations, were presented previously [18]. The donors, whose islets were included in the present study, had the following characteristics; 10 T2D donors, 5 males and 5 females, age = 66.5±2.8 years and BMI = 26.6±1.1 kg/m2 and 47 non-diabetic donors, 29 males and 18 females, age = 52.7±2.4 years and BMI = 24.8±0.6. Calpain 10 mRNA expression was analysed in islets from all T2D donors and 34 of the non-diabetic donors. Pancreases were obtained according to the Italian National regulation and processed with the approval of the regional Ethics Committee. The donor before death or her/his relatives upon admission to Intensive Care Unit (ICU) had given their willingness to donate organs and/or tissues.

### Studies of human pancreatic islets

Isolated pancreatic islets were prepared by collagenase digestion and density gradient purification [19]. After isolation, islets were cultured free floating in M199 culture medium (Sigma-Aldrich) at 5.5 mmol/l glucose concentration (basal condition) and studied within 3 days from isolation. Cell viability, measured by trypan blue exclusion, was higher than 90% in control and diabetic islets after 3 days in culture. Islet cell death was assessed by Cell Death Detection ELISA-plus assay (Roche Diagnostics, Milan, Italy) as previously described [20]. This assay evaluates cytoplasmic histone-associated DNA fragments.

### Insulin secretion study

Insulin secretion studies were performed as previously described [19]. Following a 45 min pre-incubation period at 3.3 mmol/l glucose, groups of 30 islets of comparable size were kept at 37°C for 45 min in Krebs-Ringer bicarbonate solution (KRB), 0.5% albumin, pH 7.4, containing 3.3 mmol/l glucose. At the end of this period, medium was completely removed and replaced by KRB containing either 3.3 mmol/l glucose, 16.7 mmol/l glucose, 3.3 mmol/l glucose plus 20 mmol/l arginine, or 3.3 mmol/l glucose plus 100 µmol/l glibenclamide. After additional 45 min incubation, medium was removed. The media (500 µl) were stored at −20°C until insulin concentrations were measured by immuno-radiometric assay (IRMA, Pantec Forniture Biomediche, Turin, Italy). Insulin secretion in response to the different secretagogues in islets from non-diabetic and diabetic islets is described in **Table 1**.

**Table 1:** Insulin secretion in response to glucose, arginine and glibenclamide in human non-diabetic and diabetic pancreatic islets cultured in vitro.

	Non-diabetic	Diabetic	*P*
Glucose SI	2.08±0.17	1.25±0.11	0.002
Arginine SI	1.88±0.11	1.62±0.16	0.2
Glibenclamide SI	2.07±0.18	1.55±0.19	0.1

SI is the insulin stimulation index i.e. incremental folds above baseline insulin release. Results are mean±SEM.

### Analysis of calpain-10 mRNA levels in pancreatic islets

Total RNA was extracted from human islets, after 3 days in culture, using the RNeasy Protect Mini Kit (Qiagen Inc, Valencia, CA) and quantified by absorbance at A_260_/A_280_ (ratio>1.65) nm in a Perkin-Elmer spectrophotometer, and its integrity was assessed after electrophoresis in 1.0% agarose gels by ethidium bromide staining. Human calpain-10 mRNA levels were quantified by Reverse Transcription followed by Real-Time Quantitative RT-PCR [19]. Gene-specific probe and primer pair for calpain-10 (Assays-on-demands, Assay ID Details:

Hs01550166_m1, Applied Biosystems, Foster City, CA) was used. Each sample was run in duplicates and the transcript quantity was normalized to the mRNA level of cyclophilin A (Applied Biosystems, Foster City, CA).

### Genotyping

Genomic DNA was extracted from pancreatic islets using Wizard Genomic DNA Purification kit (Promega). Two polymorphisms, SNP-43 (rs3792267) and SNP-44 (rs2975760) of the *CAPN10* gene, were genotyped using an Allelic Discrimination assay performed with an ABI 7900 system (Applied Biosystems, Foster City, CA). Primers and probes were designed using Assay by Design (Applied Biosystems); SNP-43 (G/A) forward primer 5′-GCG CTC ACG CTT GCT-3′, reverse primer 5′-CCT CAC CAA GTC AAG GCT TAG C-3′, probe-1 5′(VIC)-AAG TAA GGC GTT TGA AG-3′, probe-2 5′(FAM)-AAG TAA GGC ATT TGA AG-3′and SNP-44 (C/T) forward primer 5′-GCA GGG CGC TCA CG-3′, reverse primer 5′-CCT CAC CAA GTC AAG GCT TAG C-3′, probe-1 5′(VIC)-CCT TAC TTC GCA GCA AG-3′, probe-2 5′(FAM)-CCT TAC TTC ACA GCA AG-3′.

### Statistical methods

Differences in calpain-10 mRNA levels and insulin secretion between the different groups studied were analyzed using non-parametric Mann-Whitney test and multivariate regression analysis with adjustments for age, sex and BMI. The significance of differences in expression and insulin secretion between genotypes was analyzed using one-way ANOVA, followed by Kruskal-Wallis Z test. All *P* values were two-tailed and *P* values less than 0.05 were considered significant. Statistical calculations were performed by NCSS software (NCSS Statistical Software, Kaysville, UT).

## Results

**Figure 1:**
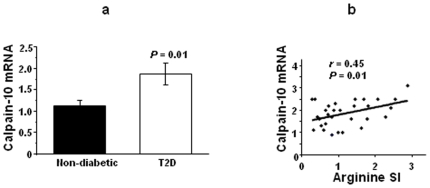
Calpain-10 mRNA levels in human pancreatic islets are influenced by type 2 diabetes (a) and correlates with arginine-stimulated insulin release in non-diabetic islets (b). SI is the insulin stimulation index i.e. incremental folds above baseline insulin release. Results are expressed as mean±SEM.

The mRNA level of calpain-10 was increased in pancreatic islets from patients with T2D compared with non-diabetic donors (1.87±0.26 n = 10 versus 1.14±0.12 n = 34; *P* = 0.01) (**Fig. 1a**). Since the diabetic donors were significantly older and had higher BMI than control donors, we further tested if these factors might also relate to calpain-10 expression in human islets using a multivariate regression analysis including age, sex, BMI and disease status as covariates. Only disease status was significantly associated with calpain-10 expression in this analysis (beta = 0.66; *P* = 0.04). Neither SNP-43 nor SNP-44 were associated with calpain-10 mRNA expression in non-diabetic donors (*P* = 0.32 and *P* = 0.58, respectively) (**Table 2**). However, there was an association between SNP-43 and glucose stimulated insulin secretion (GSIS) (G/G 0.072±0.013 n = 20, G/A 0.088±0.016 n = 13, A/A 0.10±0.026 n = 5, µU/islet/minutes; *P* = 0.022) and the insulin stimulation index (SI; incremental folds above baseline insulin release) (G/G 1.91±0.25 n = 20, G/A 2.23±0.31 n = 13, A/A 2.43±0.49 n = 5; *P* = 0.04) for a dominant model in response to 16.7 mmol/l glucose in islets from non-diabetic donors (**Table 2**). In contrast to glucose-stimulated insulin secretion, there was no effect of SNPs in the *CAPN10* gene on arginine- or glibenclamide-stimulated insulin secretion in vitro (**Table 2**). In non-diabetic donors the mRNA level of calpain-10 correlated positively with insulin release in response to arginine (*r* = 0.45; *P* = 0.015) (**Fig. 1b**) but not in response to glucose (*r* = 0.20; *P* = 0.3) or glibenclamide (*r* = 0.22; *P* = 0.26). However, the correlation between calpain-10 expression and insulin release in response to arginine was lost in T2D donors (*r* = 0.09; *P* = 0.8). Although not significant, while the correlation between calpain-10 expression and the level of apoptosis was negative in a subset of islets from non-diabetic donors (*r* = −0.31; *P* = 0.27, n = 14), the correlation was positive in a subset of islets from diabetic donors (*r* = 0.54; *P* = 0.27, n = 6).

**Table 2:** Association between SNP-43 and SNP-44 of the *CAPN10* gene and calpain-10 mRNA expression, glucose-, arginine- and glibenclamide-stimulated insulin secretion in human non-diabetic pancreatic islets cultured in vitro.

***SNP-43***	**G/G**	**G/A**	**A/A**	***P***
Calpain-10 expression	1.00±0.18 (16)	1.33±0.22 (11)	1.20±0.37 (4)	0.32
Basal insulin secretion^1^	0.034±0.0028 (20)	0.039±0.0035 (13)	0.044±0.0056 (5)	0.066
GSIS^1^	0.072±0.013 (20)	0.088±0.016 (13)	0.10±0.026 (5)	0.022^2^
Glucose SI	1.91±0.25 (20)	2.23±0.31 (13)	2.43±0.49 (5)	0.04^2^
Arginine SI	1.87±0.16 (18)	2.15±0.19 (12)	1.55±0.33 (4)	0.13
Glibenclamide SI	2.04±0.26 (19)	2.26±0.33 (12)	1.83±0.65 (3)	0.25
***SNP-44***	**T/T**	**T/C**	**C/C**	***P***
Calpain-10 expression	1.18±0.14 (26)	1.03±0.32 (5)	0.42 (1)	0.58^2^
Basal insulin secretion^1^	0.037±0.002 (30)	0.033±0.005 (6)	0.03±0.009 (2)	0.45^2^
GSIS^1^	0.084±0.011 (30)	0.06±0.024 (6)	0.05±0.04 (2)	0.27^2^
Glucose SI	2.21±0.21 (30)	1.71±0.47 (6)	1.67±0.81 (2)	0.34^2^
Arginine SI	2.00±0.14 (25)	1.52±0.28 (6)	2.15±0.48 (2)	0.09
Glibenclamide SI	2.04±0.23 (25)	1.90±0.57 (6)	2.85±0.57 (2)	0.98

Basal insulin secretion is analysed when culturing islets in 5.5 mmol/l glucose. Glucose stimulated insulin secretion (GSIS) is analysed after culturing islets in 16.7 mmol/l glucose for 45 min as described in the method section.

1 µU/islet/minutes. SI is the insulin stimulation index i.e. incremental folds above baseline insulin release. Results are mean±SEM with number of individuals shown in parentheses.

2 For a dominant model. *P* values are not corrected for multiple testing.

## Discussion

The key results from this study were that 1) calpain-10 mRNA levels were elevated in pancreatic islets of patients with type 2 diabetes and 2) there was a positive correlation between calpain-10 expression and insulin release in response to arginine in non-diabetic but not in diabetic donors.

*CAPN10* was the first gene linked to T2D risk through positional cloning (2). Though the intimate mechanisms for such linkage are not totally clear yet, implications for a role of calpain-10 in insulin action and secretion are plausible. Calpains are Ca^2+^ dependent cystein proteases that catalyze the cleavage of specific substrates and thereby regulate biological pathways. Moreover, Ca^2+^ plays an important role in insulin release from β-cells in response to secretagogues. Since calpain-10 has been implicated in the pathogenesis of T2D, it may be an important regulator of insulin secretion. Indeed, calpain-10 has been suggested to play a role in facilitating the actin reorganization required for glucose-stimulated insulin release [21] and inhibition of calpains blocks insulin secretion [13], [16]. In line with our data, calpain-10 was recently identified in human pancreatic islets and there was a positive correlation between calpain-10 levels and insulin release in response to a secretagogue cocktail in INS-1 cells [14]. Marshall et al also suggested that calpain-10 is involved in the first phase exocytosis and that it binds to the plasma membrane SNARE complex and may affect SNAP-25 proteolysis [14]. In agreement with this suggestion we observed a positive correlation between calpain-10 expression and insulin release in response to arginine in human non-diabetic islets. One could speculate that an arginine dependent increase in intracellular Ca^2+^ stimulates the activity of calpain-10, which then binds to the SNARE complex and regulates insulin release. However, the correlation between calpain-10 and insulin release in response to arginine was lost in islets from patients with T2D, where calpain-10 expression was elevated by 64% compared with non-diabetic islets. This is difficult to translate to the clinical condition because although first phase insulin secretion is characteristically lost in the very early stages of T2DM, arginine response is usually retained. Whether other factors may be operative *in vivo*, that are missing in the *in vitro* situation remains to be ascertained. The loss of correlation between calpain-10 expression and arginine-stimulated insulin secretion in diabetic donors could also be due to the small number of donors in this group. Nevertheless, we cannot exclude that the different response to arginine as a function of calpain-10 expression may reflect a true defect between diabetic and non-diabetic islets. Interestingly, calpain-10 has been suggested to induce apoptosis in response to fatty acids (16). It may, therefore, be possible that calpain-10 has a positive effect on insulin secretion of healthy islets, whereas it may enhance lipotoxic activation of apoptosis in T2D islets effecting overall insulin secreting function. Indeed, although not significant, the opposite correlations between calpain-10 expression and the level of apoptosis in islets from non-diabetic and diabetic donors support this hypothesis. However, this theory needs to be explored further in future studies.

Combinations of genetic and environmental factors influence the susceptibility to T2D. *CAPN10* was the first T2D gene to be identified by positional cloning [2] and some studies have then replicated this association. Although, we and others found associations between polymorphisms in the *CAPN10* gene and its expression in skeletal muscle and adipose tissue, the present study was unable to detect a significant association between SNP-43 and SNP-44 and calpain-10 mRNA levels in human pancreatic islets. It is indeed possible that these SNPs do not influence gene expression in islets. On the other hand, our analysis only included islets from 34 non-diabetic donors so we may not have had enough power to detect an association. Nevertheless, there was a nominal association between SNP-43 and insulin release from human islets cultured *in vitro* in response to glucose. Although, previous studies have found associations between the G/G variant and elevated insulin secretion in vivo [15], [22], the enhanced insulin secretion could represent a compensatory response to decreased insulin sensitivity. Indeed, the association between SNP-43 and insulin secretion in vivo was lost when insulin secretion was adjusted for insulin sensitivity [15]. However, one can not exclude that in vitro studies give different results compared with in vivo analysis.

In conclusion, our data suggest a contribution of calpain-10 expression on insulin secretory function in human isolated pancreatic islets. Whether the loss of an association between calpan-10 expression and arginine-stimulated insulin release documented in T2D islets reflects a specific pathophysiologic aspect will require further studies.
